# Inflammatory Biomarkers and Clinical Outcomes in Hospitalized Hemodialysis Patients with COVID-19: A Retrospective Observational Study

**DOI:** 10.3390/diagnostics16101498

**Published:** 2026-05-14

**Authors:** Oana Nicolescu, Mihaela Magdalena Mitache, Andrei Mitache, Adelina-Gabriela Niculescu, Dragos Garofil, Victor Dan Eugen Strambu, Bogdan Oancea, Marian Necula, Corneliu Ovidiu Vrancianu, Ioana Ruxandra Poiana, Adrian Radu Petru, Ana Maria Alexandra Stănescu

**Affiliations:** 1 Faculty of Medicine, Carol Davila University of Medicine and Pharmacy, 020021 Bucharest, Romania; droananicolescu@yahoo.com (O.N.); dragos.garofil@umfcd.ro (D.G.); victor.strambu@umfcd.ro (V.D.E.S.); ioana-ruxandra.poiana@umfcd.ro (I.R.P.); petru.radu@umfcd.ro (A.R.P.); alexandra.stanescu@umfcd.ro (A.M.A.S.); 2 Faculty of Medicine, University Titu Maiorescu Bucharest, Gheorghe Petrașcu 67A, 031592 Bucharest, Romania; mitache.ad@gmail.com; 3 Public Health Directorate, Avrig 72-74, District 2, 021578 Bucharest, Romania; 4 Research Institute of the University of Bucharest—ICUB, University of Bucharest, Șoseaua Panduri 90, District 5, 050663 Bucharest, Romania; adelina.niculescu@upb.ro (A.-G.N.); ovidiu.vrancianu@incdsb.ro (C.O.V.); 5 Department of Science and Engineering of Oxide Materials and Nanomaterials, National University of Science and Technology, POLITEHNICA, Splaiul Independenței 313, District 6, 060042 Bucharest, Romania; 6 Dr. Carol Davila Clinical Hospital of Nephrology, Calea Griviței 4, 010731 Bucharest, Romania; 7 National Institute of Research and Development for Biological Sciences, 296 Splaiul Independentei, District 6, 060031 Bucharest, Romania; bogdan.oancea@faa.unibuc.ro (B.O.); marian.necula@faa.unibuc.ro (M.N.); 8 Faculty of Administration and Business, University of Bucharest, Regina Elisabeta 4-12, 030167 Bucharest, Romania; 9 Doctoral School, Carol Davila University of Medicine and Pharmacy, Eroii Sanitari 8, District 5, 050474 Bucharest, Romania; 10 Academy of Romanian Scientists AOSR, Ilfov Street 3, District 5, 030167 Bucharest, Romania

**Keywords:** SARS-CoV-2, COVID-19, maintenance hemodialysis, biomarkers, inflammation, prognosis, chronic kidney disease, dialysis management, mortality, survival

## Abstract

**Background/Objectives**: Maintenance hemodialysis patients are particularly vulnerable to severe acute respiratory syndrome coronavirus 2 (SARS-CoV-2) infection. This study aimed to evaluate clinical outcomes and identify admission laboratory biomarkers associated with in-hospital mortality in hospitalized hemodialysis patients with coronavirus disease 2019 (COVID-19). **Methods**: We conducted a retrospective observational study including 130 adult hemodialysis patients with confirmed SARS-CoV-2 infection. Clinical characteristics and admission laboratory parameters were analyzed in relation to in-hospital outcomes using comparative, multivariable logistic regression, and receiver operating characteristic (ROC) curve analyses. **Results**: The overall in-hospital mortality rate was 34.6%. The median age of the cohort was 66 years, with 64.6% male patients. Non-survivors showed significantly higher levels of inflammatory and tissue-injury markers, including C-reactive protein (CRP) (*p* < 0.001) and lactate dehydrogenase (LDH) (*p* < 0.001), together with lower serum albumin (*p* < 0.001), platelet count (*p* < 0.001), and lymphocyte levels (*p* = 0.03). In multivariable analysis, cardiovascular disease, respiratory disease, dyspnea, and ambulatory origin were independently associated with mortality. ROC analysis identified platelet count as the best individual predictor (area under the curve [AUC] = 0.767). An exploratory composite risk score demonstrated excellent discriminative performance (AUC = 0.902). **Conclusions**: Admission inflammatory and hematological biomarkers are strongly associated with adverse outcomes in hospitalized hemodialysis patients with COVID-19. The integration of clinical and laboratory parameters into a composite risk score may improve early risk stratification and support clinical decision-making in this high-risk population.

## 1. Introduction

Emerging in Wuhan, China, in December 2019, coronavirus disease 2019 (COVID-19) rapidly spread to the USA and Europe by January 2020, primarily through international travel and community transmission [1]. In Romania, as in other countries, the pandemic evolved in successive waves characterized by different viral variants and heterogeneous clinical impact, with increasing pressure on healthcare systems across successive peaks (Table 1) [2,3,4].

In this context, inflammatory and hematological biomarkers measured at hospital admission have been increasingly investigated as predictors of disease severity and mortality in COVID-19 [5,6,7]. Markers such as C-reactive protein (CRP), lactate dehydrogenase (LDH), and lymphocyte counts have consistently been associated with adverse outcomes across multiple studies [8,9,10,11,12,13,14]. In addition, derived indices integrating inflammatory and nutritional status, including the CRP-to-albumin ratio (CAR), neutrophil-to-lymphocyte ratio (NLR) [15], and fibrinogen-to-albumin ratio (FAR), have gained attention as potential prognostic tools.

Beyond these commonly used indices, a growing body of evidence highlights the prognostic relevance of additional composite inflammatory markers that capture distinct aspects of immune dysregulation and systemic inflammation. The platelet-to-lymphocyte ratio (PLR) and monocyte-to-lymphocyte ratio (MLR) have been associated with inflammatory burden and adverse outcomes in patients with chronic kidney disease (CKD) and those undergoing hemodialysis, reflecting the interplay between innate immune activation and lymphocyte-mediated immune suppression [16,17]. More complex indices, such as the systemic immune-inflammation index (SII), which integrates neutrophil, platelet, and lymphocyte counts, have demonstrated prognostic value in predicting adverse clinical outcomes and vascular complications in dialysis populations [18]. In parallel, the prognostic nutritional index (PNI), combining serum albumin and lymphocyte count, provides a composite measure of nutritional and immunological status and has been shown to independently predict mortality in hemodialysis patients [19]. Despite these promising findings, the comparative and combined utility of these emerging biomarkers remains insufficiently explored, particularly in the context of severe acute respiratory syndrome coronavirus 2 (SARS-CoV-2) infection in dialysis-dependent populations.

Among patients with chronic illnesses, individuals with CKD undergoing maintenance hemodialysis represent one of the most vulnerable populations. Their increased susceptibility arises from a combination of uremia-associated immune dysfunction affecting both innate and adaptive immunity, chronic systemic inflammation, advanced age, and a high prevalence of cardiovascular and metabolic comorbidities, including diabetes mellitus [20,21,22]. These baseline alterations may amplify the host response to SARS-CoV-2 infection, predisposing dialysis patients to severe disease, rapid clinical deterioration, and increased mortality [21,23,24].

In addition to immune dysfunction, SARS-CoV-2 infection has been linked to cardiovascular complications, including cardiac injury, thrombotic and thromboembolic events, and dysregulation of glucose and lipid metabolism, likely driven by endothelial damage, hypercoagulability, and systemic inflammation [3,25,26,27]. These mechanisms are particularly relevant in CKD patients, in whom pre-existing endothelial dysfunction and vascular fragility may exacerbate COVID-19 severity and contribute to worse outcomes [26].

However, despite increasing evidence in the general population, data specifically addressing the integrated prognostic value of inflammatory and hematological biomarkers in hemodialysis patients remain limited and heterogeneous. Real-world studies in dialysis cohorts have primarily focused on clinical predictors such as age, symptoms, and comorbidities, with less emphasis on composite biomarker-based risk stratification [3]. Furthermore, regional data from Eastern Europe are scarce, despite differences in healthcare system adaptations and pandemic dynamics.

Based on these considerations, we hypothesized that admission inflammatory and hematological biomarkers, including both conventional parameters (CRP, LDH) and derived indices (CAR, FAR, NLR, PLR, MLR, SII, and PNI), are significantly associated with in-hospital mortality in hemodialysis patients with COVID-19. Specifically, we postulated that markers reflecting systemic inflammation and immune dysregulation would correlate with increased mortality risk, while composite indices integrating inflammatory and nutritional status would provide enhanced prognostic discrimination in this high-risk population. In addition, we aimed to evaluate whether extended inflammatory indices (NLR, PLR, MLR, SII, and PNI) offer complementary or superior prognostic value compared to conventional biomarkers.

Therefore, the aim of this study was to evaluate clinical outcomes, dialysis management adaptations, and admission laboratory parameters associated with mortality in hemodialysis patients hospitalized with SARS-CoV-2 infection.

## 2. Materials and Methods

### 2.1. Study Design and Population

This retrospective observational study included 130 adult patients with CKD undergoing maintenance hemodialysis who were diagnosed with SARS-CoV-2 infection during hospitalization and admitted to a nephrology unit between March 2020 and April 2021. This period spans the early pandemic and subsequent waves in Romania, before widespread vaccination. During this time, SARS-CoV-2 circulation was initially dominated by early pandemic lineages, including the original Wuhan-Hu-1 strain and variants carrying the D614G mutation. By early 2021, the Alpha (B.1.1.7) variant became the predominant circulating strain, while later variants such as Delta were not yet dominant during this interval. In addition, a small number of cases admitted after March 2021 were analyzed separately.

During the study period, COVID-19 vaccination in Romania was initiated in late December 2020 and gradually expanded in early 2021. Therefore, most patients included in this cohort were likely unvaccinated, particularly those admitted during the first and second pandemic waves. Limited vaccination coverage was present during the third wave and thereafter.

The study population was defined based on the following inclusion and exclusion criteria:

Inclusion criteria: Adult patients (≥18 years) with CKD undergoing maintenance hemodialysis, admitted to the nephrology unit between March 2020 and April 2021, with confirmed SARS-CoV-2 infection by real-time reverse transcription polymerase chain reaction (RT-PCR).

Exclusion criteria: Patients admitted outside the study period; patients not receiving maintenance hemodialysis; absence of RT-PCR confirmation of SARS-CoV-2 infection; and cases with incomplete or missing essential clinical data.

All consecutive patients meeting these criteria during the study period were included.

This study was designed as a prognostic analysis restricted to hemodialysis patients diagnosed with COVID-19. Non-infected patients were not included as a comparison group in order to maintain a homogeneous study population and to avoid confounding related to differences in disease exposure, clinical management, and inflammatory status, which could have affected the interpretability of biomarker–outcome associations.

### 2.2. Patient Selection and Cohort Construction

A total of 500 patients undergoing maintenance hemodialysis were evaluated during 2020. Of these, 100 patients (20.0%) were excluded at the initial screening stage, including 25 patients on peritoneal dialysis (5.0%) and 75 patients evaluated for kidney transplantation (15.0%), leaving 400 patients eligible for further assessment. Among these, 215 patients (53.8%) did not develop COVID-19 during the monitored interval and were therefore excluded. Of the remaining 185 patients with a history of COVID-19, 85 (45.9%) presented outside the predefined study interval and were excluded. Finally, 100 patients met all eligibility criteria and had complete data, forming the 2020 cohort. During the period from 1 January to 30 April 2021, a total of 140 patients were assessed. Of these, 29 patients (20.7%) were excluded (4 on peritoneal dialysis and 25 evaluated for transplantation), leaving 111 eligible patients. Among them, 30 patients (27.0%) developed COVID-19 during the monitored interval and fulfilled all inclusion criteria. All had complete datasets and were included in the 2021 cohort. Overall, the final study population consisted of 130 patients, including 100 patients from the 2020 cohort and 30 patients from the 2021 cohort.

Patients were stratified by the admission period corresponding to the national pandemic waves in Romania. Because viral genomic sequencing was unavailable, wave-based classification was used as a temporal proxy, reflecting changes in circulating SARS-CoV-2 lineages and clinical context rather than confirmed variant attribution.

### 2.3. Data Collection

Data were retrospectively extracted from medical records. Collected variables included demographic characteristics, clinical features at admission, major comorbidities (particularly cardiovascular disease and diabetes mellitus), and in-hospital outcomes (discharge or death). Dialysis-related information was limited to confirmation of maintenance hemodialysis status and documentation of dialysis access–related complications or modality changes when explicitly reported in the medical records.

Biological parameters were obtained at hospital admission for all available cases and further analyzed in relation to clinical evolution, with particular attention to unfavorable outcomes, including severe disease progression and mortality. Cases with missing laboratory or clinical data were included only in analyses for which complete data were available.

### 2.4. Laboratory Assessment

COVID-19 diagnosis was confirmed by RT-PCR testing of nasopharyngeal swabs.

Laboratory investigations were performed at hospital admission and included complete blood count, inflammatory markers, and biochemical parameters. Hematological variables comprised total leukocyte count, neutrophil count, and lymphocyte count. Inflammatory status was assessed using CRP, fibrinogen, and serum ferritin levels. LDH was used as a marker of tissue injury and metabolic stress. Results are presented as mean ± standard deviation (SD).

### 2.5. Outcomes

The primary outcome was in-hospital mortality. Secondary outcomes included discharge status and the need for dialysis regimen adjustments during acute infection. In-hospital mortality was defined as death occurring during the index hospitalization.

### 2.6. Statistical Analysis

Statistical analyses were performed using R software (version 4.5.2; R Foundation for Statistical Computing, Vienna, Austria). Continuous variables were assessed for distribution and are presented as median and interquartile range (IQR) due to non-normal distribution. Categorical variables are expressed as frequencies and percentages. Comparisons between survivors and non-survivors were performed using the Mann–Whitney U test for continuous variables and the chi-square or Fisher’s exact test for categorical variables, as appropriate. Correlation analyses were conducted using Spearman’s rank correlation coefficient (ρ). A two-tailed *p*-value < 0.05 was considered statistically significant.

Receiver operating characteristic (ROC) analyses were performed to evaluate the discriminative performance of individual biomarkers, with AUCs and 95% confidence intervals (CI) calculated using DeLong’s method. Optimal cut-off values were determined using the Youden index, and corresponding sensitivity and specificity were reported.

An exploratory multivariable logistic regression model was developed to generate a composite risk score using selected non-redundant predictors. Variables were standardized prior to modeling. Model discrimination was assessed using ROC analysis, while internal validation was performed using bootstrap resampling. Calibration was evaluated by comparing predicted and observed probabilities across risk deciles and visualized using a calibration plot. Model fit was assessed using the Hosmer–Lemeshow test, and multicollinearity was examined using variance inflation factors.

## 3. Results

### 3.1. Clinical Outcomes

**Figure 1:**
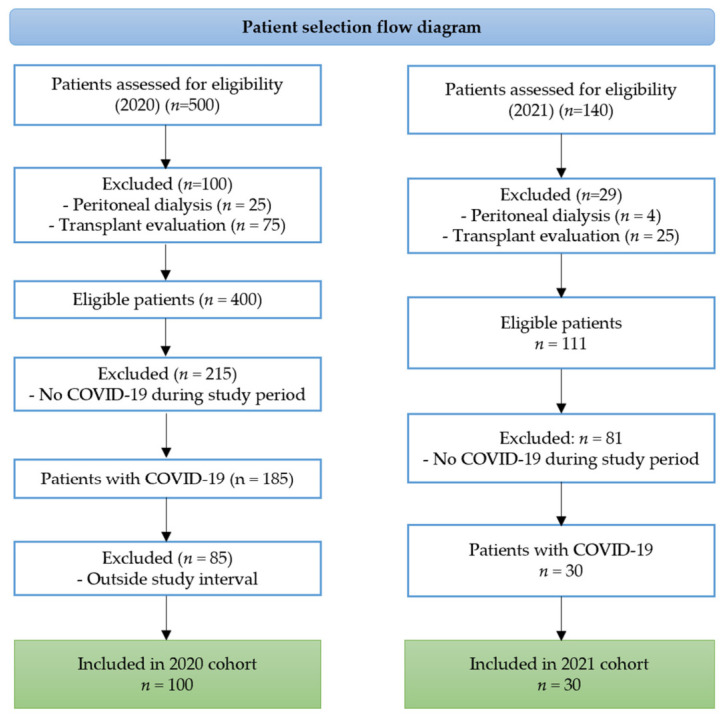
Flow diagram of patient selection and inclusion in the final study cohort. The diagram illustrates the screening process of patients undergoing maintenance hemodialysis during the study period (2020–2021), including exclusion criteria and the final inclusion of patients with confirmed severe acute respiratory syndrome coronavirus 2 (SARS-CoV-2) infection and complete clinical data; COVID-19, coronavirus disease 19.

A total of 130 patients met all inclusion criteria and were included in the final analysis. These patients were selected from a larger cohort of individuals undergoing maintenance hemodialysis during the study period, following predefined inclusion and exclusion criteria. The patient selection process, including screening and exclusion steps, is illustrated in Figure 1.

Clinical outcomes of the study population are presented in Table 2, with an overall in-hospital mortality rate of 34.6%. Unfavorable clinical evolution was more frequently observed in patients with cardiovascular comorbidities and diabetes mellitus. Specifically, patients with cardiovascular conditions and diabetes mellitus showed lower survival rates (64.0% and 59.7%, respectively).

When stratified by admission period corresponding to national pandemic waves (Table 3), most patients were hospitalized during the second wave (July–December 2020). The highest mortality rate was observed during this period (38.2%). Mortality during the first wave was 31.4%, whereas a lower mortality rate was observed during the third wave (20.0%), although the sample size was small (*n* = 10). Only a small number of admissions were recorded after March 2021 (*n* = 8), and outcomes in this period should therefore be interpreted cautiously.

**Figure 2:**
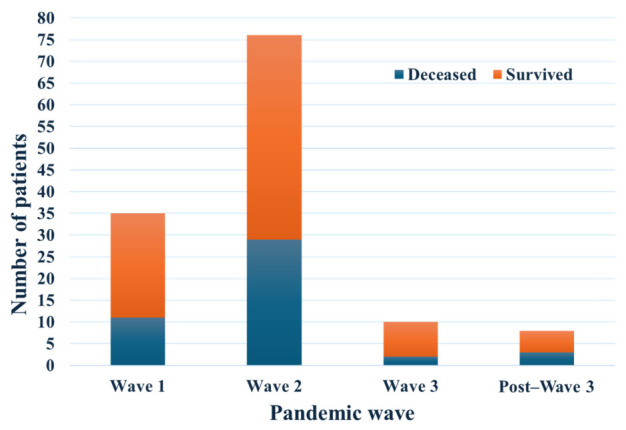
Distribution of survivors and deceased patients across national pandemic waves, defined by admission period.

Figure 2 illustrates the distribution of survivors and deceased patients across pandemic waves. The highest number of admissions occurred during the second wave, which also accounted for the largest number of deaths. Fewer admissions were observed during the first and third waves and in the post–wave 3 period, with correspondingly smaller numbers of deaths.

### 3.2. Baseline Characteristics According to In-Hospital Outcome

The baseline demographic and clinical characteristics of the study population stratified by in-hospital outcome are presented in Table 4. The median age did not differ significantly between survivors and non-survivors, and no significant differences were observed in sex distribution. However, cardiovascular disease and dyspnea were significantly more frequent among non-survivors. In addition, patients with fatal outcomes were more often admitted from hospital settings.

### 3.3. Predictors of In-Hospital Mortality

To identify independent predictors of in-hospital mortality, a multivariable logistic regression analysis was performed (Table 5). Dyspnea, cardiovascular disease, and respiratory comorbidities were significantly associated with increased mortality risk, while hypertension and ambulatory admission were associated with lower odds of death.

In addition, several admission laboratory parameters demonstrated independent prognostic significance. Higher levels of inflammatory and tissue injury markers, including CRP, LDH, and composite indices such as the CAR and FAR, were significantly associated with increased mortality risk. Conversely, higher albumin levels, lymphocyte count, platelet count, and PNI were associated with lower odds of in-hospital death.

Other hematological indices, including NLR, PLR, MLR, and SII, did not show statistically significant associations in the multivariable model.

### 3.4. Biological Profile of Unfavorable Evolution

Admission laboratory parameters were analyzed in relation to clinical outcomes. Overall, patients exhibited elevated inflammatory markers, including CRP, fibrinogen, ferritin, and LDH, alongside hematological alterations such as leukocytosis, neutrophilia, and relatively low lymphocyte levels (Table 6).

Comparative analysis between survivors and non-survivors (Table 7) revealed significantly higher levels of CRP and LDH in deceased patients, accompanied by lower serum albumin and platelet counts. In addition, white blood cell counts were elevated, and lymphocyte levels were reduced in non-survivors.

Among derived inflammatory indices, CAR and FAR were significantly higher in deceased patients (*p* < 0.001), whereas NLR, although elevated, did not reach statistical significance (*p* = 0.253).

**Figure 3:**
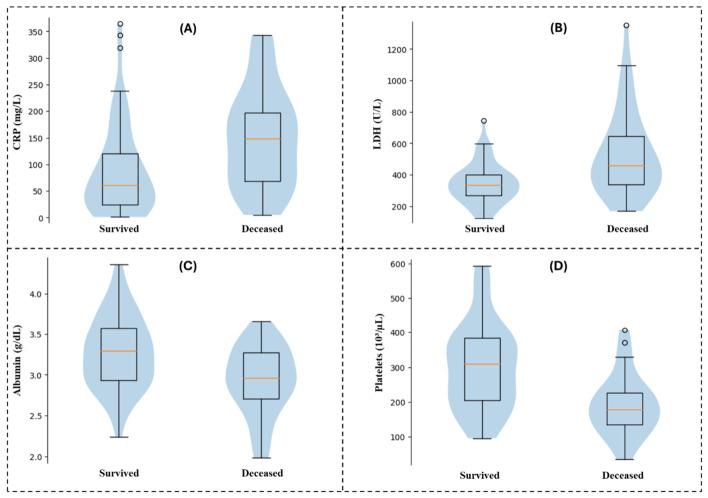
Distribution of key inflammatory and hematologic biomarkers according to clinical outcome. Violin plots with overlaid boxplots illustrating admission levels of (**A**) C-reactive protein (CRP), (**B**) lactate dehydrogenase (LDH), (**C**) serum albumin, and (**D**) platelet count in hospitalized hemodialysis patients with COVID-19, stratified by in-hospital outcome (survived vs. deceased). Within each boxplot, the central yellow line represents the median, the box indicates the interquartile range (IQR), whiskers represent 1.5 × IQR, and circles denote outliers.

These group-level differences are further illustrated by the distributions shown in Figure 3, where CRP and LDH values are shifted toward higher ranges in deceased patients, whereas albumin and platelet counts tend to be lower.

### 3.5. Correlation Analysis

**Figure 4:**
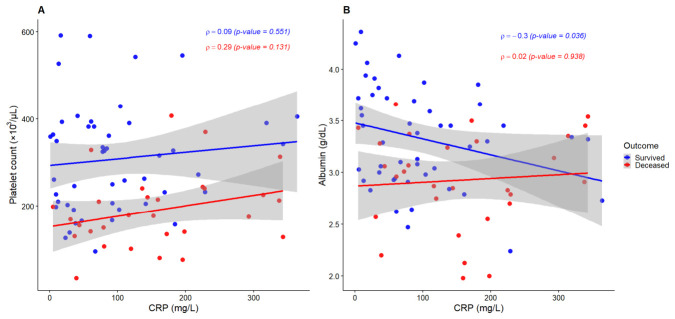
C-reactive protein (CRP) versus (**A**) platelet count and (**B**) serum albumin by clinical outcome. Spearman correlation coefficients (r) and *p*-values are shown for each outcome group. Solid lines represent linear regression trends, while shaded grey areas indicate 95% confidence intervals.

Correlation analyses were performed to explore relationships between inflammatory and hematological parameters (Figure 4). No significant association was observed between CRP and platelet count in either survivors (ρ = 0.09, *p* = 0.55) or deceased patients (ρ = 0.29, *p* = 0.13). In contrast, CRP showed a modest but statistically significant inverse correlation with serum albumin among survivors (ρ = −0.30, *p* = 0.036), while no significant association was identified in deceased patients (ρ = 0.02, *p* = 0.94).

These findings support the interplay between systemic inflammation and nutritional status and provide a biological rationale for evaluating their combined prognostic performance in subsequent ROC and multivariable analyses.

### 3.6. ROC Analysis

**Figure 5:**
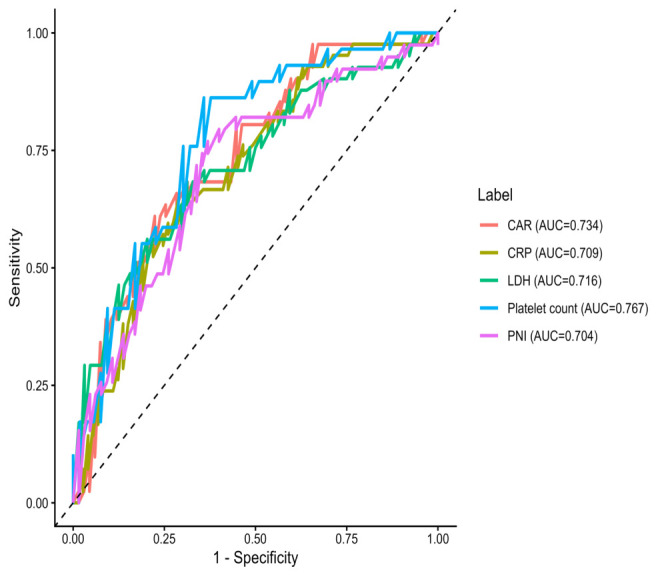
Receiver operating characteristic (ROC) curves for selected biomarkers with statistically significant discriminative ability for predicting in-hospital mortality; CRP, C-reactive protein; LDH, lactate dehydrogenase; CAR, CRP-to-albumin ratio; PNI, prognostic nutritional index. The dashed diagonal line represents the line of no discrimination (AUC = 0.5).

The discriminative performance of individual biomarkers was evaluated using ROC analysis (Figure 5 and Table 8). For clarity, only biomarkers with statistically significant discriminative ability were included in the ROC plot. Platelet count showed the highest performance (AUC = 0.767), followed by CAR (AUC = 0.734), LDH (AUC = 0.716), CRP (AUC = 0.709), and PNI (AUC = 0.704), all demonstrating moderate predictive ability.

In contrast, traditional inflammatory ratios such as NLR (AUC = 0.538) and MLR (AUC = 0.492) showed limited discriminative capacity.

Optimal cut-off values were determined using the Youden index, with corresponding sensitivity and specificity detailed in Table 8. Overall, most biomarkers exhibited moderate discriminative performance. Importantly, several parameters demonstrated statistically significant predictive ability, including platelet count (AUC = 0.767, *p* < 0.001), CAR (AUC = 0.734, *p* < 0.001), LDH (AUC = 0.716, *p* < 0.001), CRP (AUC = 0.709, *p* < 0.001), PNI (AUC = 0.704, *p* < 0.001), and albumin (AUC = 0.699, *p* < 0.001).

In contrast, other indices such as NLR, MLR, SII, and PLR did not reach statistical significance, indicating limited discriminative performance in this cohort. These findings highlight the variability in prognostic utility across individual biomarkers and support the use of combined or composite approaches for improved risk stratification.

### 3.7. Composite Risk Score

To improve prognostic accuracy, an exploratory multivariable logistic regression model was developed to generate a composite risk score. The model incorporated LDH, CAR, and platelet count, selected to represent complementary biological domains including tissue injury, systemic inflammation, and hematological response.

**Figure 6:**
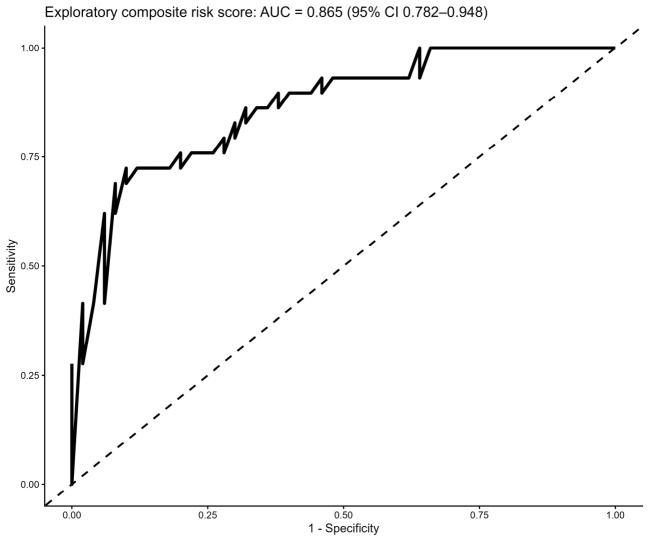
Receiver operating characteristic (ROC) curve for the exploratory composite risk score predicting in-hospital mortality. The model demonstrated excellent discriminative performance (area under the curve [AUC] = 0.902, 95% confidence interval [CI] 0.83–0.973), substantially outperforming individual biomarkers. The dashed diagonal line represents the line of no discrimination (AUC = 0.5).

The composite score demonstrated excellent discriminative performance, with an AUC of 0.902 (95% CI 0.83–0.973), substantially outperforming all individual biomarkers (Figure 6).

**Figure 7:**
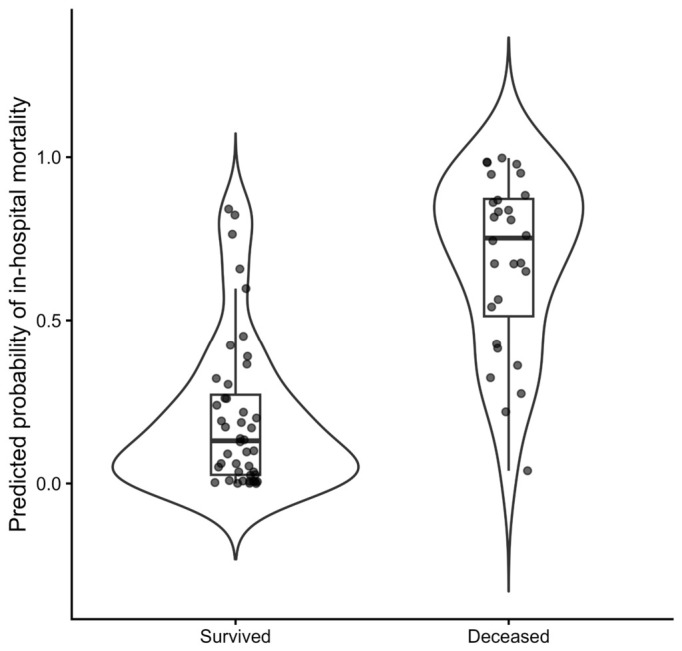
Distribution of the predicted probabilities derived from the composite risk score, stratified by in-hospital outcome (survived vs. deceased). The model shows clear separation between groups, indicating strong discriminative ability at the individual level. Dots represent individual patient-level predicted probabilities overlaid on violin plots with embedded boxplots.

The distribution of predicted probabilities showed clear separation between survivors and non-survivors (Figure 7), indicating strong individual-level discrimination.

**Figure 8:**
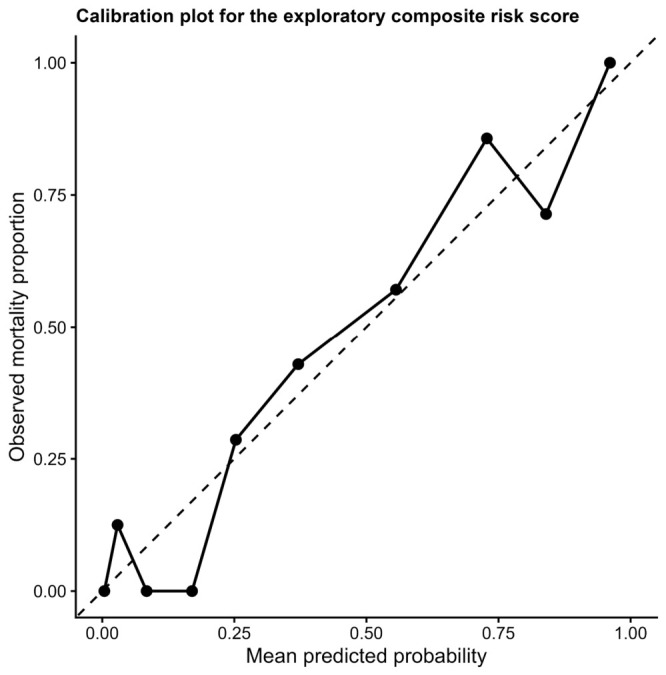
Calibration plot of the exploratory composite risk score. The relationship between predicted and observed in-hospital mortality is shown across risk deciles. The dashed line represents perfect calibration. The model demonstrates good agreement between predicted probabilities and observed outcomes. The dashed diagonal line represents perfect calibration between predicted and observed probabilities.

Calibration analysis demonstrated good agreement between predicted and observed mortality across risk deciles (Figure 8), supporting the internal validity of the model.

## 4. Discussion

In this retrospective cohort, the observed mortality rate was 34.6%, highlighting the profound vulnerability of patients receiving maintenance hemodialysis when infected with SARS-CoV-2. This figure is substantially higher than mortality rates reported in the general COVID-19 population and aligns closely with international reports describing mortality rates of approximately 30–40% among hospitalized dialysis patients [23,28]. Similar outcomes have been described in both single-center and registry-based studies, confirming the disproportionate effects of the pandemic on dialysis patients, compared with the general population.

Patients undergoing maintenance hemodialysis present unique pathophysiological mechanisms that predispose them to severe infectious outcomes. CKD is characterized by persistent immune dysregulation, endothelial dysfunction, and a proinflammatory and prothrombotic milieu [22,25], all of which may exacerbate SARS-CoV-2–induced inflammatory response and lead to a more rapid clinical deterioration [29]. In addition, the high prevalence of cardiovascular disease and diabetes mellitus further amplifies vulnerability [3,26,27], a pattern also observed in our cohort, where these comorbidities have been repeatedly associated with unfavorable outcomes.

In line with these observations, our baseline analysis demonstrated that cardiovascular disease was significantly more frequent among non-survivors, while respiratory comorbidities and dyspnea at admission were also markedly associated with mortality. In contrast, no significant differences were observed in age or sex distribution between outcome groups, suggesting that clinical severity and comorbidity burden, rather than demographic factors alone, drive prognosis in this population.

Moreover, the present study highlights the prognostic relevance of inflammatory and biochemical markers measured at hospital admission. In our cohort, deceased patients exhibited significantly higher levels of CRP, LDH, ferritin, and fibrinogen, along with leukocytosis, neutrophilia, and relative lymphopenia, indicating a pronounced systemic inflammatory response and multiorgan involvement. This pattern is consistent with immune dysregulation and tissue injury mechanisms widely described in severe SARS-CoV-2 infection. In dialysis patients, these alterations may be further amplified by baseline immune dysfunction and chronic inflammatory status, contributing to rapid clinical deterioration and poor outcomes.

These findings are further supported by the integration of clinical and biological variables in multivariable analysis, where cardiovascular and respiratory comorbidities, as well as dyspnea at admission, emerged as independent predictors of in-hospital mortality, reinforcing their clinical relevance beyond univariate associations.

Importantly, the expanded multivariable model incorporating laboratory biomarkers confirmed that inflammatory and nutritional parameters, including CRP, LDH, albumin, platelet count, and composite indices such as CAR and FAR, also contribute independently to mortality risk, highlighting the multidimensional nature of disease severity in this population.

These findings are in line with previously reported international studies. From a clinical perspective, these results support the routine use of readily available inflammatory and hematological parameters at hospital admission for early risk stratification in hemodialysis patients with COVID-19. In cohorts from China, Italy, and the United States, elevated inflammatory markers and hematological abnormalities were strongly associated with COVID-19 severity and mortality [8,9,10]. Meta-analyses have consistently identified CRP, LDH, ferritin, and lymphocyte depletion as robust predictors of unfavorable outcomes in hospitalized COVID-19 patients, including those with CKD [12].

In addition to individual biomarkers, derived inflammatory indices have gained increasing attention as integrated markers of systemic inflammation and clinical severity. In our cohort, both the CAR and the FAR were significantly higher in non-survivors, supporting their potential role as prognostic indicators in patients undergoing maintenance hemodialysis with COVID-19. These ratios reflect the combined effects of inflammation and nutritional status, both of which are critical determinants of outcomes in this high-risk population.

In contrast, although the NLR was elevated in non-survivors, the difference did not reach statistical significance, suggesting that its prognostic value may be more limited in this specific clinical context. This finding is partially consistent with previous studies and meta-analyses in CKD populations, where elevated NLR has been associated with increased mortality, although with variable predictive performance [30,31]. This observation is further supported by the ROC analysis, where several hematological indices, including NLR, PLR, MLR, and SII, did not demonstrate statistically significant discriminative ability.

Importantly, this discrepancy may reflect a limitation of single-axis inflammatory markers in capturing the complex and multidimensional pathophysiology of dialysis patients, where baseline immune dysfunction and chronic inflammation may reduce the discriminatory capacity of isolated hematological ratios.

The observed inverse correlation between CRP and serum albumin among survivors further highlights the interplay between systemic inflammation and diminished physiological reserve in hemodialysis patients with COVID-19. Although this association was not evident among deceased patients, the overall pattern reinforces the role of albumin as a marker reflecting both inflammatory burden and baseline vulnerability in this high-risk population.

Notably, the absence of a similar correlation in non-survivors may suggest a loss of physiological regulatory mechanisms in advanced disease stages, where systemic inflammation becomes uncoupled from compensatory responses, further contributing to poor outcomes.

Beyond individual biomarkers, our findings support the concept that severe COVID-19 in dialysis patients reflects an exaggerated inflammatory response. Hyperferritinemia and elevated fibrinogen levels further support the presence of a hyperinflammatory state resembling cytokine storm syndromes described in severe SARS-CoV-2 infections from early pandemic waves [32]. Such immune dysregulation may be particularly detrimental in dialysis patients, whose impaired immune surveillance limits viral clearance while favoring uncontrolled inflammation.

Importantly, while individual biomarkers demonstrated only moderate discriminative ability (AUC values generally below 0.80), their combined use in a multivariable framework resulted in a substantial improvement in prognostic performance. Consistent with ROC findings, only a subset of biomarkers, including platelet count, CAR, LDH, CRP, and PNI, showed statistically significant discriminative performance, further supporting their selection for clinical interpretation and model development.

The exploratory composite risk score integrating LDH, CAR, and platelet count achieved excellent discrimination (AUC = 0.902), clearly outperforming all individual predictors. This finding underscores the complementary nature of these biomarkers, which capture distinct but interrelated biological domains, including tissue injury, systemic inflammation, and hematological response. Importantly, the selection of these variables was supported by their independent contribution in multivariable analysis, further strengthening the biological and clinical plausibility of the proposed model.

Furthermore, the clear separation of predicted probabilities between survivors and non-survivors and the good agreement observed in the calibration analysis indicate that the model is not only discriminative but also clinically reliable in estimating individual risk.

These results suggest that multimarker approaches may overcome the limitations of single biomarkers and provide a more robust and clinically applicable framework for early risk stratification in complex populations such as patients undergoing maintenance hemodialysis.

From an organizational and epidemiological perspective, the temporal distribution of admissions and deaths across pandemic waves in our cohort showed a clear concentration during the second pandemic wave, which accounted for the highest number of hospitalizations and deaths. This pattern likely reflects both increased community transmission and the heightened vulnerability of dialysis patients during the early pandemic period, when therapeutic strategies and healthcare workflows were still evolving.

However, the variability in mortality across waves should also be interpreted in the context of changing clinical management, evolving treatment protocols, and potential differences in viral variants, which may have influenced disease severity and outcomes independently of patient-related factors.

Comparative studies from different regions consistently demonstrate elevated mortality in dialysis cohorts, reinforcing the concept that dialysis dependence represents a major independent risk factor for adverse COVID-19 outcomes [28,33]. The convergence of findings across diverse healthcare systems suggests that intrinsic patient-related factors, rather than regional practice differences alone, drive this excess risk.

Vaccination represented a critical turning point in the COVID-19 pandemic, markedly altering the clinical landscape of SARS-CoV-2 infection in dialysis patients. Recent cohort studies have reported a substantial reduction in COVID-19-related mortality following vaccine rollout, despite all-cause mortality remaining high due to competing comorbidities [9,24].

In this context, the identification of reliable prognostic models becomes even more relevant, as risk stratification may guide targeted interventions and optimize resource allocation in both pandemic and post-pandemic settings.

This study has several limitations that should be acknowledged. First, its retrospective design limits causal inference, and the moderate sample size may restrict generalizability. The absence of a non-infected dialysis control group precludes direct comparison of outcomes attributable solely to SARS-CoV-2 infection. Additionally, the exploratory nature of the composite risk model requires external validation in independent cohorts before clinical implementation, as overfitting cannot be fully excluded despite internal validation procedures.

Despite these limitations, the present study provides a comprehensive clinical and biological characterization of hospitalized hemodialysis patients with COVID-19. Importantly, it demonstrates that integrating routinely available biomarkers into composite models may significantly enhance prognostic accuracy, offering a practical and scalable approach for clinical decision-making in high-risk populations.

## 5. Conclusions

SARS-CoV-2 infection is associated with substantial in-hospital mortality among patients undergoing maintenance hemodialysis, confirming the marked vulnerability of this population during acute infectious outbreaks. Admission inflammatory and hematological biomarkers, including CRP, LDH, ferritin, fibrinogen, leukocyte count, and lymphocyte levels, were significantly associated with adverse clinical outcomes.

Importantly, while individual biomarkers demonstrated only moderate predictive performance, their integration into a composite risk model substantially improved discrimination. The exploratory risk score combining LDH, CAR, and platelet count achieved excellent predictive accuracy (AUC = 0.902), highlighting the added value of multimarker approaches that capture complementary biological pathways.

These findings support the clinical utility of combining routinely available laboratory parameters for early risk stratification in hemodialysis patients with COVID-19. Such approaches may facilitate timely decision-making and more personalized management strategies in this high-risk population. Further prospective validation is warranted to confirm the applicability of these models in clinical practice.

## Figures and Tables

**Table 1:** Pandemic waves in Romania from January 2020 to December 2021. Adapted from an open-access source [2].

Pandemic Wave	Wave 1		Wave 2		Wave 3		Wave 4	
	Start	End	Start	End	Star	End	Start	End
Period	Jan 2020	Jun 2020	Jul 2020	Dec 2020	Jan 2021	Mar 2021	Sep 2021	Nov 2021
Dominant Variant	Wuhan-Hu-1 (NCBI RefSeq: NC_045512.2)		Clade variant S: D614G		Alpha (B.1.1.7)		Delta (B.1.617.2)	

NCBI = National Center for Biotechnology Information; RefSeq = Reference Sequence.

**Table 2:** Clinical outcomes of dialysis patients infected with SARS-CoV-2.

Outcome	Number of Patients (*n*)	Percentage (%)
Discharged	84	64.6
Deceased	45	34.6
Incomplete clinical data	1	0.8
Total	130	100

**Table 3:** Clinical outcomes of dialysis patients according to the SARS-CoV-2 pandemic wave.

Pandemic Wave	Wave Time Interval in Romania	Deceased (*n*)	Total (*n*)	Mortality (%)
Wave 1	January–June 2020	11	35	31.4
Wave 2	July–December 2020	29	76	38.2
Wave 3	January–March 2021	2	10	20.0
Post-Wave 3	After March 2021	3	8	37.5

**Table 4:** Baseline clinical characteristics according to in-hospital outcome.

Variable	Survivors (*N* = 83)	Non-Survivors (*N* = 45)	*p*-Value
Age (years)	65 (54–71)	69 (55–78)	0.13
Male sex, *n* (%	56 (67%)	26 (58%)	0.40
Hypertension, *n* (%)	66 (80%)	28 (62%)	0.057
Diabetes, *n* (%)	37 (45%)	24 (53%)	0.40
Cardiovascular disease, *n* (%)	37 (45%)	31 (69%)	0.014
Respiratory disease, *n* (%)	11 (13%)	13 (29%)	0.054
Multimorbidity ≥2, *n* (%)	79 (95%)	45 (100%)	0.30
Fever, *n* (%)	17 (20%)	15 (33%)	0.20
Dyspnea, *n* (%)	19 (23%)	26 (58%)	<0.001
Fatigue, *n* (%)	14 (17%)	5 (11%)	0.50
Hospital admission, *n* (%)	46 (55%)	37 (82%)	0.005
Length of stay (days)	12 (4–19)	13 (8–17)	0.70

Values are presented as median (IQR) or number (percentage). Comparisons were performed using the Mann–Whitney U test for continuous variables and the chi-square or Fisher’s exact test for categorical variables, as appropriate.

**Table 5:** Multivariable logistic regression analysis for in-hospital mortality.

Variable	Comparison	OR (95% CI)	*p*-Value
Age (years)	per year increase	1.01 (0.99–1.04)	0.30
Female sex	vs. male	1.49 (0.71–3.21)	0.30
Hypertension	yes vs. no	0.44 (0.19–0.95)	0.041
Diabetes	yes vs. no	1.40 (0.69–2.96)	0.40
Cardiovascular disease	yes vs. no	2.64 (1.30–6.06)	0.011
Respiratory disease	yes vs. no	2.52 (1.08–6.69)	0.039
Multimorbidity (≥2)	vs. <2	6.61 (0.35–125)	0.20
Fever	yes vs. no	1.88 (0.85–4.42)	0.12
Dyspnea	yes vs. no	4.36 (2.13–10.3)	<0.001
Fatigue	yes vs. no	0.64 (0.19–1.75)	0.40
Ambulatory origin	vs. hospital	0.29 (0.11–0.62)	0.004
Length of stay (days)	per day	1.00 (0.96–1.04)	>0.9
CRP	per unit increase, log-scaled	2.048 (1.369–3.190)	<0.001
LDH	per unit increase, log-scaled	1.993 (1.193–3.746)	<0.001
Albumin	per unit increase, log-scaled	0.421 (0.252–0.661)	<0.001
Lymphocyte count	per unit increase, log-scaled	0.550 (0.320–0.930)	0.022
Platelet count	per unit increase, log-scaled	0.101 (0.026–0.315)	<0.001
NLR	per unit increase, log-scaled	1.066 (0.689–1.627)	0.759
CAR	per unit increase, log-scaled	2.251 (1.476–3.588)	<0.001
FAR	per unit increase, log-scaled	1.768 (1.119–2.964)	0.024
PLR	per unit increase, log-scaled	0.612 (0.315–1.042)	0.151
MLR	per unit increase, log-scaled	0.722 (0.294–1.279)	0.914
SII	per unit increase, log-scaled	0.705 (0.372–1.171)	0.21
PNI	per unit increase, log-scaled	0.520 (0.330–0.810)	<0.001

CRP, C-reactive protein; LDH, lactate dehydrogenase; NLR, neutrophil-to-lymphocyte ratio; CAR, CRP-to-albumin ratio; FAR, fibrinogen-to-albumin ratio; PLR, platelet-to-lymphocyte ratio; MLR, monocyte-to-lymphocyte ratio; SII, systemic immune-inflammation index; PNI, prognostic nutritional index.

**Table 6:** Biological markers associated with unfavorable clinical evolution in dialysis patients with COVID-19.

Biological Marker	Mean ± SD
Lymphocytes (10^3^/µL)	4.52 ± 13.95
Neutrophils (10^3^/µL)	28.84 ± 31.08
LDH (U/L)	415.1 ± 211.0
Ferritin (ng/mL)	802.45 ± 473.72
Fibrinogen (mg/dL)	511.24 ± 151.79
CRP (mg/L)	110.28 ± 91.58

LDH, lactate dehydrogenase; CRP, C-reactive protein.

**Table 7:** Admission laboratory parameters according to in-hospital outcome.

Parameter	Survivors		Deceased		*p*-Value *
	Median	IQR	Median	IQR	
CRP (mg/L)	61	24–120	148.18	68.09–197.33	<0.001
LDH (U/L)	335	269.25–402	457.67	337–647	<0.001
Albumin (g/dL)	3.295	2.93–3.58	2.96	2.71–3.27	<0.001
Platelets (×10^3^/µL)	316	205.5–390	178	136–226	<0.001
White blood cells (×10^3^/µL)	9.25	6.65–12.88	12.83	10.54–15.60	0.001
Lymphocytes (×10^3^/µL)	1.60	1.10–2.35	1.20	0.91–1.72	0.03
NLR	5.10	2.79–28.26	8.25	4.98–10.87	0.253
CAR	0.39	0.12–0.72	0.99	0.60–1.72	<0.001
FAR	1.08	0.69–1.57	1.43	1.13–2.07	<0.001

IQR, interquartile range; * *p*-values derived from non-parametric tests (Mann–Whitney U), given non-normal distribution of biomarkers; CRP, C-reactive protein; LDH, lactate dehydrogenase; NLR, neutrophil-to-lymphocyte ratio; CAR, C-reactive protein-to-albumin ratio; FAR, fibrinogen-to-albumin ratio.

**Table 8:** ROC analysis for all evaluated parameters.

Biomarker/Index	*N*	AUC (95% CI)	*p*-Values	Optimal Cut-Off	Sensitivity	Specificity
Platelet count	82	0.767 (0.662–0.873)	<0.001	245.000	0.862	0.642
CAR	108	0.734 (0.637–0.83)	<0.001	41.422	0.610	0.776
LDH	105	0.716 (0.613–0.82)	<0.001	384.580	0.683	0.688
CRP	115	0.709 (0.612–0.806)	<0.001	111.500	0.643	0.712
PNI	104	0.704 (0.599–0.809)	<0.001	39.500	0.769	0.631
Albumin	112	0.699 (0.601–0.796)	<0.001	3.375	0.881	0.400
FAR	92	0.633 (0.518–0.748)	0.024	120.202	0.829	0.439
Lymphocyte count	110	0.627 (0.518–0.735)	0.022	1.680	0.744	0.493
PLR	80	0.593 (0.466–0.721)	0.151	218.084	0.833	0.400
SII	74	0.585 (0.452–0.719)	0.21	3927.162	0.759	0.467
NLR	95	0.538 (0.419–0.658)	0.529	4.875	0.784	0.431
MLR	63	0.492 (0.343–0.64)	0.914	0.227	0.739	0.425

AUC, area under the curve; *N*, sample size; CRP, C-reactive protein; LDH, lactate dehydrogenase; NLR, neutrophil-to-lymphocyte ratio; CAR, CRP-to-albumin ratio; FAR, fibrinogen-to-albumin ratio; PLR, platelet-to-lymphocyte ratio; MLR, monocyte-to-lymphocyte ratio; SII, systemic immune-inflammation index; PNI, prognostic nutritional index.

## Data Availability

The original contributions presented in this study are included in the article. Further inquiries can be directed to the corresponding author.

## Abbreviations

The following abbreviations are used in this manuscript:

SARS-CoV-2	severe acute respiratory syndrome coronavirus 2
COVID-19	coronavirus disease 19
ROC	receiver operating characteristic
CRP	C-reactive protein
LDH	lactate dehydrogenase
SD	standard deviation
AUC	area under the curve
CAR	CRP-to-albumin ratio
NLR	neutrophil-to-lymphocyte ratio
FAR	fibrinogen-to-albumin ratio
PLR	platelet-to-lymphocyte ratio
MLR	monocyte-to-lymphocyte ratio
CKD	chronic kidney disease
SII	systemic immune-inflammation index
PNI	prognostic nutritional index
RT-PCR	real-time reverse transcription polymerase chain reaction
IQR	interquartile range
CI	confidence interval
